# Data-driven curation process for describing the blood glucose management in the intensive care unit

**DOI:** 10.1038/s41597-021-00864-4

**Published:** 2021-03-10

**Authors:** Aldo Robles Arévalo, Jason H. Maley, Lawrence Baker, Susana M. da Silva Vieira, João M. da Costa Sousa, Stan Finkelstein, Roselyn Mateo-Collado, Jesse D. Raffa, Leo Anthony Celi, Francis DeMichele

**Affiliations:** 1 IDMEC, Instituto Superior Técnico, Universidade de Lisboa, Lisbon, Portugal; 2 Beth Israel Deaconess Medical Center, Boston, MA USA; 3 RAND Corporation, Santa Monica, CA USA; 4 Massachusetts Institute of Technology, Cambridge, MA USA; 5 Rush University Medical Center, Chicago, IL USA; 6 Harvard T.H. Chan School of Public Health, Boston, MA USA; 7 Landmark Health, Huntington Beach, CA USA

**Keywords:** Outcomes research, Data mining, Data processing

## Abstract

Analysis of real-world glucose and insulin clinical data recorded in electronic medical records can provide insights into tailored approaches to clinical care, yet presents many analytic challenges. This work makes publicly available a dataset that contains the curated entries of blood glucose readings and administered insulin on a per-patient basis during ICU admissions in the Medical Information Mart for Intensive Care (MIMIC-III) database version 1.4. Also, the present study details the data curation process used to extract and match glucose values to insulin therapy. The curation process includes the creation of glucose-insulin pairing rules according to clinical expert-defined physiologic and pharmacologic parameters. Through this approach, it was possible to align nearly 76% of insulin events to a preceding blood glucose reading for nearly 9,600 critically ill patients. This work has the potential to reveal trends in real-world practice for the management of blood glucose. This data extraction and processing serve as a framework for future studies of glucose and insulin in the intensive care unit.

## Abstract (summary)

**Table:** 

Measurement(s)	Blood Glucose • insulin (human)
Technology Type(s)	glucose analyzer • glucometer • Electronic Medical Record
Factor Type(s)	glucose readings • insulin inputs
Sample Characteristic - Organism	Homo sapiens

Machine-accessible metadata file describing the reported data: 10.6084/m9.figshare.13564187

## Background & Summary

There are relatively few randomized controlled trials that study glycemic control in the intensive care unit (ICU) setting. This is due to the complexity of studying large populations and standardizing protocols for managing glucose across different medical centers. Current clinical practice guidelines are general and recommend avoiding both hypoglycemia and hyperglycemia in critically ill patients^1^.

Retrospective analysis of real-world data can potentially reveal valuable insights into specific ranges of glycemic targets^2^ which may provide a survival advantage for certain populations of critically ill patients. The currently available large real-world data sets are not suitable in their unprocessed form to answer these important clinical questions. Here we present a comprehensive approach to the extraction and processing of insulin treatments and blood glucose readings of critically ill patients from electronic medical records.

The following analysis demonstrates how to convert database queries of unprocessed glucose and insulin values into a clinically validated and reproducible dataset. It highlights the challenges of interpreting the variable of blood glucose level while it is measured in two different and contemporaneous methods. It proposes how to stratify the multiple different types of insulin administered in the ICU. Also, it shows how to create time-series records at the individual patient level of measured blood glucose versus the dose of administered insulin. The process of pairing each administration of insulin to a corresponding glucose value allows for the potential creation of a model that shows both how glucose is managed in the real-world and also how patients respond to this therapy in a per-patient time series for each specified ICU admission in the dataset.

The present paper used real patient blood glucose and administered insulin values to create these individual time series records. It was employed the MIMIC-III v.1.4 database^3^, a publicly available dataset, contains over 58,000 hospital admissions from approximately 38,600 adults. Identifying these patients through traditional manual chart review would be impractically time intensive. The primary challenges of our project were (1) matching a dose of regular insulin to the specific glucose value that triggered the treatment and (2) identification of values that were likely erroneously entered for both blood glucose entries and insulin entries. The reason erroneous entries exist is that documentation in electronic medical records is subject to human error and can be limited due to other clinical priorities in the ICU. For that purpose, it was necessary to create, interrogate, and iteratively refine rules to pair and align each insulin event to the preceding blood glucose. The rules were derived from a consensus between a group of clinicians, including an internist, an intensivist, and an endocrinologist. The clinicians derived the rules from physiologic and pharmacologic standards.

Data scientists first sorted and identified specific subgroups of patients, as defined by clinical experts. This then allowed clinical experts to identify erroneous entries of blood glucose and insulin doses. This identification step requires consideration of the type of insulin, timing of administration, timing of glucose measurement, institutional nursing practices, and glucose values. For example, clinicians are not only necessary for defining abnormal values, but also identifying appropriate parameters for linking glucose to insulin administration based on clinical protocols; and identifying scenarios when “outlier” glucose readings may represent true values and not errors. This step of data curation is crucial before any machine learning can be performed^4–6^.

**Fig. 1:**
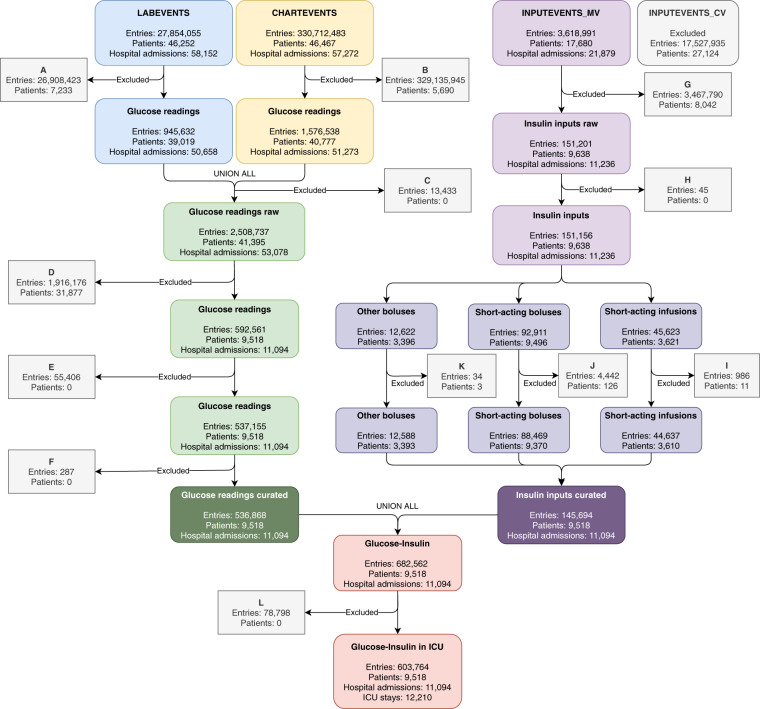
Inclusion criteria for data curation process. Refer to the Methods section to follow and read this figure entirely.

Figure 1 outlines the experimental workflow used to curate glucose readings and insulin inputs, and posterior merging to create one data subset for future analysis. Access to the complete code to reproduce this data curation process is provided to allow others to continue to generate insights related to the management of blood glucose in the ICU. Also, the codes of the validation assays for this data subset are provided.

Through sharing this data curation process of paired glucose and insulin values, it is publicly available to provide a reference framework for extracting and matching real-world hospital data. We provide a detailed methodology to assist future research around glycemic control in the ICU using real-world data. Thus, this study can also be used as a starting point for retrospective analysis of outcomes in the ICU, inform future clinical trial designs, and generate new treatment approaches^7,8^. Also, we highlight that strong collaboration between clinical experts and data scientists must exist from the beginning of these projects to improve the validity of data representation – for example, the pairing of insulin and glucose readings.

## Methods

The following subsections describe the extraction and processing of the data required to understand glucose control during an ICU stay. The primary categories of data chosen for extraction were glucose readings and insulin inputs. All ICU stays with at least one event of glucose reading and/or insulin input were included. It is suggested to read these subsections along with Fig. 1.

### Data extraction and Pre-processing

#### Glucose data collection

In the ICU setting, patient blood glucose values are measured using either laboratory chemistry analyzers or bedside fingerstick glucometers^9^. In MIMIC-III, laboratory analyzer glucose values are recorded in the LABEVENTS and CHARTEVENTS tables (Fig. 1). Fingerstick glucometer measurements are only recorded in CHARTEVENTS. Glucose values were recorded across 10 different Item IDs in MIMIC-III (Table 1), 7 Item IDs within the CHARTEVENTS table, and 3 Item IDs within the LABEVENTS table. The raw number of instances of glucose recordings was 2,508,737 within a population of 41,395 patients after merging the Item IDs referred to in Table 1 and removing glucose values that were zero or recorded as an error. These errors occurred during the measurement in the hospital (https://mimic.physionet.org/mimictables/chartevents/). Erroneous entries are indicated in the ERROR column from the CHARTEVENTS table as not recorded or recorded as 1 (criteria *A* and *B* Fig. 1).

**Table 1:** Item ID’s related to glucose values identified in MIMIC-III and the associated method for reading glycemia.

Table	Item ID	Description	Analytical technique	Instances	
				Raw	Curated
CHARTEVENTS	807	Fingerstick Glucose	Fingerstick	431,010	1,124
	811	Glucose (70–105)	Fingerstick	374,668	1,363
	1529	Glucose	Fingerstick	283,795	1,446
	3744	Blood Glucose	Fingerstick	530	—
	3745	Blood Glucose	Laboratory analyzer	2,639	—
	225664	Glucose finger stick	Fingerstick	246,001	218,888
	220621	Glucose (serum)	Laboratory analyzer	154,525	97,596
	226537	Glucose (whole blood)	Fingerstick	70,231	58,356
LABEVENTS	50809	Glucose - Chemistry - Blood	Laboratory analyzer	196,591	35,170
	50931	Glucose - Blood Gas - Blood	Laboratory analyzer	748,747	122,925
			**Total**	**2,508,737**	**536,868**
			**Patients**	**41,395**	**9,518**

Then after joining the information of the LABEVENTS and CHARTEVENTS tables, the remaining null values, where no result was recorded, were removed (criterion *C* Fig. 1). Posteriorly, patients who did not receive any insulin were excluded (criterion *D* Fig. 1). Duplicate values were removed when identical readings with the same timestamp and glucose values appeared in both LABEVENTS and CHARTEVENTS (criterion *E* Fig. 1).

To further curate the glucose readings, the following considerations were taken into account:

- Glucose measured from the laboratory analyzer is considered more accurate than the fingerstick glucometer, especially at higher glucose levels. However, in the clinical setting fingerstick glucometer measurements are used frequently and insulin can be dosed based on fingerstick measurements alone. Therefore, both methods of glucose measurement were included in the extraction scripts.
- Glucose measurements were extracted with the following rules (criterion *F* in Fig. 1):
  - All glucose values ≥1000 mg/dL were removed. These values were removed because they were above the limit of accurate measurement for the laboratory analyzer used at Beth Israel Deaconess Medical Center (BIDMC).
  - If a sample was taken from serum and the value was <1000 mg/dL, then that glucose value was included.
  - If a sample was taken with the fingerstick method and the value was <500 mg/dL, then that glucose value was included. It was determined that in this dataset, all fingerstick values above this threshold were simply recorded as “500”. These values were removed because they are above the limit of accurate measurement for the fingerstick glucometer analytical method used at BIDMC.

The time stamps recorded for glucose readings are based on charting by nursing staff in the ICU. Due to other clinical priorities and patient care issues, there may be errors in the time stamp as entered by the nurses. To account for this, the following assumptions were made:

- Sometimes the STORETIME (time listed by nurses for checking glucose) was recorded before the CHARTTIME (the time when the actual data entry occurred). In that case, the STORETIME timestamp was considered to be the time when the glycemic check occurred. Otherwise, the CHARTTIME timestamp was maintained as the time of glycemic check (criterion *F* of Fig. 1).

The inclusion criteria described above resulted in 536,868 instances of unique glucose measurements within a population of 9,518 patients who received insulin during their admission to the hospital. The number of raw and curated instances of glucose measurements for each Item ID are also displayed in Table 1.

The number of entries that have values above the limit of accurate measurement for each analytical method, were not significant. Only 144 entries for blood samples analyzed in the laboratory were removed (0.06% of entries remaining after applying criterion *F* in Fig. 1). In the case of bedside fingerstick samples, 146 entries were removed (0.05% of entries remaining after applying criterion *F* in Fig. 1). Presumed delays in the recording of the glucose readings occurred in 44,926 cases, which represents 8.4% of the total glucose readings (*n* = 536,868; criterion *F* in Fig. 1). The median of this delay was 25 min.

Once all of these criteria were considered, the values were merged with the curated subset containing the insulin inputs (Fig. 1). Once being merged, the remaining glucose readings occurring outside the ICU stay were removed (criterion *L* in Fig. 1).

#### Insulin data collection

In the ICU, insulin is administered as a medication to control hyperglycemia. There are many forms of insulin and they are classified according to the duration of their effect, short (4–8 hours), intermediate (10–12 hours), or long (12–24 hours). Insulin is also classified based on the route of administration including intravenous continuous infusion, intravenous bolus, or subcutaneous bolus. Infusions and boluses in MIMIC-III, including type, are recorded in the INPUTEVENTS_CV (*CareVue* by Philips) and INPUTEVENTS_MV (*MetaVision* by iMDSoft) tables (Fig. 1). *CareVue* covers the period from 2001–2008, while *MetaVision* covers the years from 2008–2012.

Since *MetaVision* provides richer features (*e.g*.: insulin type), only *MetaVision* records were curated as shown in Fig. 1. The events with the flag rewritten and/or related to other inputs were removed (criterion *G* in Fig. 1). Rewritten entries are incorrect inputs that were not delivered to patients (https://mimic.physionet.org/mimictables/inputevents_mv/). Thus, the rates and amounts described were excluded. Insulin administration events were recorded in 6 different item IDs in MIMIC-III, each corresponding to a different type of insulin. The raw number of instances of insulin administration was 151,201 within a population of 9,638 patients.

Insulin administration events that did not have an associated ICU stay ID (*i.e*.: blanks or null values) were removed as well (criterion *H* in Fig. 1). These events occurred outside of the ICU setting.

It is important to note that insulin infusions and insulin boluses are recorded differently. Infusions are recorded when the infusion is started, when the rate is changed and when the infusion is discontinued. The duration of a certain rate of infusion is used to calculate the amount of continuous infusion of insulin administered within a time window. This is different from insulin boluses which are documented as separate events.

For further curation, some considerations were applied to the insulin inputs to remove outliers or to be able to capture as much data as possible:

- When the current infusion rate is not recorded, this is interpreted to mean that the rate has not changed since the last data input, which is recorded in the ORIGINALRATE column (criterion I in Fig. 1).
- For regular insulin boluses, values < 18.0 U represent 99% of all values. For infusions, rates < 29.8 U/hour represent 99% of all entries. Values above the 99th percentile were determined by clinical experts to be erroneous and excluded (criterion J for boluses and criterion I for infusions in Fig. 1). In all cases, values ≤ 0 were excluded (criteria I, J, and K in Fig. 1).

Once all these criteria were considered, the insulin data were incorporated with the curated subset containing the glucose readings (Fig. 1). Afterward, each regular insulin input, whether administered as a bolus or a change in the rate of an infusion, was aligned to a glucose reading as explained below.

After merging the curated information, the longest length of stay (LOS) was about 102 days and the shortest less than 1 day. The latter accounts for 0.1% of the included ICU stays (12,210 ICU stays in total). On average, these patients have a LOS of 12.0 ± 13.0 days and a median of 7 days. However, 1 day is the most frequent length of stay, which represents 10.7% of included ICU stays. Shorter stays or equal to one week gather over 50.0% of included ICU admissions.

#### Glucose readings and insulin inputs time alignment

Associating the glucose readings with insulin inputs was the next step. This task aimed to align each regular insulin event with a glucose measurement. From here on, we shall only focus on the regular insulin bolus administrations since these are the most common insulin input in MIMIC-III (Table 2). According to standardized ICU protocols, regular insulin administration should be preceded by a blood glucose measurement.

**Table 2:** Item ID’s related to insulin infusions or boluses identified in INPUTEVENTS_MV table. It furthers contains the number of instances before and after data curation and how many instances were lost during that process.

Item ID	Description	Acting type	Instances		Loss (%)
			Raw	Curated	
223258	Insulin Regular	Short-acting	108,237	104,484	3.5
223262	Insulin Humalog	Short-acting	30,341	28,622	5.7
223260	Insulin Glargine	Long-acting	8,641	8,626	<0.1
223259	Insulin NPH	Intermediate-acting	3,383	3,369	<0.1
223257	Insulin 70/30	Intermediate-acting	441	437	<0.1
223261	Insulin Humalog 75/25	Intermediate-acting	158	156	<0.1
		**Total**	**151,201**	**145,694**	**3.6**
		**Patients**	**9,638**	**9,518**	**1.2**

The goal was to link each insulin dose with the nearest glucose measurement. For this complex task, the following rules were implemented:

- **Rule 1**: A glucose reading should precede a regular insulin administration by up to 90 minutes. This basis for this time window was derived from the diabetic ketoacidosis guidelines which recommend measuring glucose values every 60 minutes while receiving an insulin infusion^10^. An additional 30 minutes were added, 90 minutes in total, to this interval to account for the time it may take for providers to register the event. These time intervals are within the recommendations^11^.
- **Rule 2**: When a regular insulin event was not preceded, but instead followed, by a blood glucose measurement, this glucose reading was paired with the regular insulin administration if they were recorded within 90 minutes of each other.
- **Rule 3**: Sometimes a regular insulin infusion/bolus appeared between 2 blood glucose measurements. In this case, the higher glucose value was paired with the regular insulin entry as long as they were entered within 90 minutes of each other.
- **Rule 4**: When a regular insulin bolus occurred very close to a regular insulin infusion rate, it was assumed that the patient was given a bolus and then commenced on an infusion. Both regular insulin entries were paired with the preceding blood glucose measurement, or the posterior glucose reading in case its value was higher than the preceding blood glucose and was entered within 90 minutes of the insulin dose.
- **Rule 5**: No glucose values below 90 mg/dL were paired with a subsequent regular insulin bolus or infusion. No clinician will treat this low of a blood glucose value with a regular insulin bolus or infusion.

Based on time-stamp data and proposed rules described in methods, 110,011 out of 145,678 (75.5%) insulin events were paired with a corresponding glucose measurement. The assumptions gathered in rule one paired 86,913 insulin events to a preceding glucose reading, which is 79% of the total alignments (*n* = 110,011, Table 3). It is followed by the second rule (8,841 occurrences), fourth rule (8,683 occurrences), and third rule (5,574 occurrences). The occurrences for the fifth rule were not counted being that this rule has an exclusion purpose instead of including a pair of glucose-insulin.

**Table 3:** Overview of insulin that were gathered and paired with a glucose reading after applying the proposed rules.

Administration	Action type	Patients	Insulin inputs			
			Total inputs	Inputs ICU	Paired	Not paired (%)
Infusion	Short	3,610	44,637	44,631	34,659	9,972 (22.3)
Bolus	Short	9,370	88,469	88,460	71,455	17,005 (19.2)
	Intermediate	680	3,962	3,962	964	2,998 (75.7)
	Long	2,908	8,626	8,625	2,933	5,692 (66.0)
	**Total**	**9,518**	**145,694**	**145,678**	**110,011**	**35,667 (24.5)**

In Table 3 the percentage of not paired refers to the number of inputs registered at the ICU that were not aligned. The total inputs column includes data before admission, during a stay, and after discharge from the ICU. Sixteen insulin inputs (0.01% of curated insulin inputs) were excluded due to timestamps indicating that the insulin was administered outside the time recorded for ICU length of stay (criterion *L* in Fig. 1).

### Assays for technical validation

Several assays and analyses were done to provide evidence for decisions made during our data curation process. This further increases the validity and transparency of the rules proposed in this work.

#### Symmetry

Excess kurtosis and skewness were estimated to characterize the empirical distribution of glucose readings by testing method. Skewness was estimated using the Fisher-Pearson coefficient as detailed in the documentation of the SciPy library in Python^12^.

#### Assessment of fingerstick and lab analyzer agreement

**Fig. 2:**
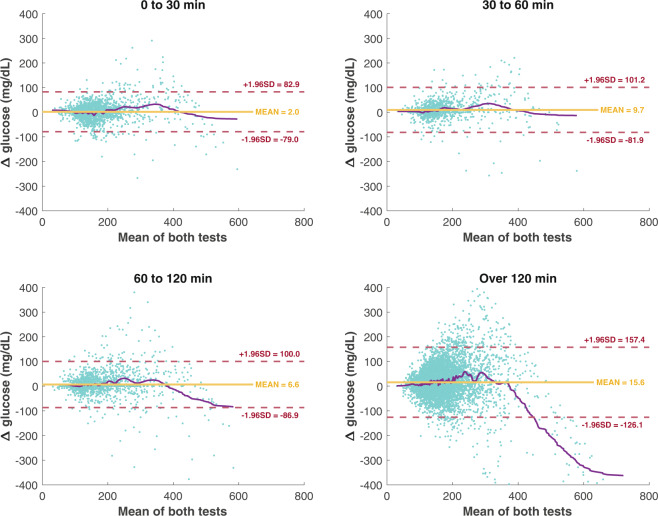
Agreement between both glucose readings methodologies. Bland-Altman plots of agreement between both glucose readings methods at 4 different time-gaps (∆ θ) or the time difference between a fingerstick reading and a sample analyzed in the chemistry lab (*n* = 11,904 paired readings). Each graph plots the mean value between a fingerstick reading and a lab analyzer sample (*x* axis), and Δ glucose (*y* axis). Horizontal lines delimit the mean difference or estimated bias (yellow continuous line) and the upper and lower agreement limits (red dashed lines) that highlight standard deviation (SD) of Δ glucose. The purple continuous line plots the smoothed values (LOWESS) of Δ glucose.

Assessment of the agreement between fingerstick and lab analyzed glucose measurements was done by identifying the first fingerstick glucose test during the ICU stay and pairing it with the lab analyzed test closest temporally to this fingerstick test. These pairs were stratified by the duration of time between the two measurements and resulted in 11,904 pairs of fingerstick – lab analyzer readings. A series of Bland-Altman plots^13^ were used to assess the agreement between the two testing types as a function of the time between the tests (Fig. 2). The graphs are further complemented with a locally weighted scatter plot smooth (LOWESS) for Δ glucose as the mean of the paired fingerstick-lab analyzed values increases.

#### Sample collection: comparison between pairing criteria

Independent samples were gathered to analyze and compare the different insulin-glucose pairing criteria discussed in this work. A pair composed of the first bolus of short-acting insulin and the corresponding preceding glucose reading were obtained per ICU stay for the three different cohorts aforementioned: (a) when a preceding glucose reading was recorded within 90 minutes of an insulin event, (b) when a preceding glucose reading was recorded within 60 minutes of the insulin event, and (c) when no pairing rules were applied.

The difference between glucose readings in scenarios **A** and **C**; and between **B** and **C** were estimated (Δ glucose [mg/dL]). These estimated values were analyzed for hypothesis testing. They were further grouped depending on the temporal difference between an insulin event and previous glycemic check (Δ*θ* [minutes]): [0 30), [30 60), [60 90), [90 120), [120 21,176).

We compared the glycemic check before the first insulin-event as a function of these temporal differences. A paired Wilcoxon rank test was used to assess the null hypothesis that the median difference in glucose measurements (scenario **C** vs **A** or **B**) was zero. Statistical significance was assessed at the 0.05 level, and all tests were two-sided.

#### Regression analysis

Lastly, we assessed if the frequency of fingerstick vs laboratory tests was associated with any admission, demographic, or clinical patient features. A multivariable logistic regression model was fit to identify covariates with an ICU admission having a higher number of bedside fingerstick readings over samples analyzed in the laboratory. Covariates were patient’s age at admission, ICU admission type, ethnicity, gender, SOFA score, and diabetes condition.

In the case of the categorical covariates of ethnicity and admission type, some categories were merged to form a more populated category. The admission types EMERGENCY (*n* = 9,504) and URGENT (*n* = 143) were merged into an EMERGENCY category and compared to elective admissions. For ethnicity, the categories WHITE - BRAZILIAN, WHITE - EASTERN EUROPEAN, WHITE - OTHER EUROPEAN, and WHITE – RUSSIAN were grouped in the WHITE category and compared to all other ethnicities.

For elderly patients >90 years old (*n* = 406), the age of these patients was imputed to 91.4 years (the median age among this group of patients, as age is otherwise withheld in MIMIC-III for regression analysis (https://mimic.physionet.org/mimictables/patients/). Regression analysis was performed on MATLAB version R2019b (The MathWorks, Inc.).

## Data Records

When the curated glucose values were separated by method of measurement, 47.6% were measured with laboratory equipment and 52.4% were measured with the bedside glucometer (Table 4). The mean value of glucose for the laboratory analyzer was 143.8 with a standard deviation of ±64.2 mg/dL. The mean value of glucose for the fingerstick glucometer was 151.3 with a standard deviation of ±59.3 mg/dL. Further, excess kurtosis and skewness values indicate that these readings do not have a normal distribution. Large kurtosis values indicate that these still have large tail sizes, this value is larger in the samples for the laboratory analyzer. This is also confirmed with positive skewness (skewed right), which is also detailed in Table 4.

**Table 4:** Frequencies and distribution of glucose observations for the included patients.

	Laboratory Analyzer	Fingerstick Glucometer	Combined
Percentage (%)	47.6	52.4	100.0
Observations	255,691	281,177	536,868
Mean glucose (mg/dL)	143.8 ± 64.3	151.3 ± 59.3	147.8 ± 61.8
Median glucose(mg/dL)	129.0	138.0	134.0
Excess kurtosis	23.6	4.9	15.0
Skewness	3.5	1.8	2.7
Minimum value (mg/dL)	4.0	0.1	0.1
Maximum value (mg/dL)	999.0	499.0	999.0
Patients	9,517	9,486	9,518

The instances of insulin administration were curated to exclude insulin administered outside of the ICU and values which were zero or clinically impossible. This resulted in 145,694 instances of insulin administration within a population of 9,518 patients. The majority of curation was performed on short-acting insulin. 3.5% of regular insulin values were excluded and 5.7% of Humalog insulin was excluded. These results are summarized in Table 2. Notably, after the glucose values and insulin administration values were curated separately the population of patients in each group was exactly equal to 9,518.

Table 5 further shows the distribution of insulin based on the method of administration before and after curation. Both the raw and curated data have a majority of insulin administrations given as short-acting insulin (91.1–90.8%) and of the short-acting type, most are administered in boluses (69.8% vs. 69.4%).

**Table 5:** Frequency of infusions and boluses events in INPUTEVENTS_MV table before and after data curation. It also contains the percentages to the total of inputs or to the total amount of boluses.

Administration	Instances		% of Boluses (raw – curated)
	Raw (%)	Curated (%)	
Bolus	105,559 (69.8)	101,057 (69.4)	100.0
Subcutaneous	96,204 (63.6)	91,756 (63.0)	91.1–90.8
Intravenous	9,355 (6.2)	9,301 (6.4)	8.9–9.2
Infusions	45,642 (30.2)	44,637 (30.6)	—
**Total**	**151,201**	**145,694**	

### Structure

A copy of each data subset has been uploaded to PhysioNet^14^ (read Usage Notes). The created subsets are two *.csv files named glucose_insulin_ICU and glucose_insulin_pair. Alternatively, these data subsets can be created and downloaded into other file extensions (*e.g.:* JSON, etc.) with the aid of the notebooks deposited on the associated PhysioNet project^15^ (read Usage notes and Code availability).

The data subsets consist of time series files that includes all the curated entries of glucose readings and insulin inputs. The file glucose_insulin_ICU.csv gathers the non-paired entries (16 columns); while the file glucose_insulin_pair.csv (21 columns) gathers the paired entries under scenario A (read Methods).

### Description of fields

***Common in both glucose_insulin_ICU.csv and glucose_insulin_pair.csv***^15^

SUBJECT_ID

It is a unique identifier for an individual patient.

HADM_ID

Represents a single patient’s admission to the hospital.

ICUSTAY_ID

Unique identifier for a single patient’s admission to the ICU.

LOS_ICU_days

Length of stay in days.

first_ICU_stay

True if it is the first admission to the ICU for a single hospital admission.

TIMER

Gathers the timestamps for either the STARTTIME for a single insulin input or the GLCTIMER for a single glucose reading. It is used to order chronologically the events during a hospital admission.

STARTTIME

A timestamp that depicts when the administration of an insulin event started.

INPUT

Dose for a single bolus of insulin in U.

INPUT_HRS

Insulin infusion rate in U/hr.

ENDTIME

A timestamp that specifies when an insulin input stops or an infusion rate was changed.

INSULINTYPE

Acting type of insulin: short, intermediate, or long (Table 2).

EVENT

Specifies whether the bolus of insulin was subcutaneous (BOLUS_INYECTION), or intravenous (BOLUS_PUSH), or if the insulin was infused (INFUSION).

INFXSTOP

Indicates when an infusion of insulin was stopped.

GLCTIMER

A timestamp that depicts when a glycemic check was done.

GLC

Glycemia value in mg/dL.

GLCSOURCE

Reading method for a glycemic check: fingerstick (FINGERSTICK) or lab analyzer (BLOOD).

***Specific to glucose_insulin_pair.csv***^15^

GLC_AL

Glycemia value in mg/dL for a paired glucose reading with a single insulin input. This value should match with the value in GLC of a preceding glucose reading according to the rule applied for this pairing case.

GLCTIMER_AL

A timestamp that depicts when a glycemic check was done for a paired glucose reading. This value should match with the timestamp in GLCTIMER of a preceding glucose reading according to the rule applied for this pairing case.

GLCSOURCE_AL

Reading method for a glycemic check that was paired with an insulin input. This value should match with the GLCSOURCE value of a preceding glucose reading according to the rule applied for this pairing case.

RULE

The rule applied for pairing a single insulin input with a preceding glucose reading.

Repeated

Indicates whether the associated glucose reading in this entry was paired with a subsequent insulin input charted in this table. These entries aid to identify and verify which glucose readings were paired. The users have the option to remove this entry if it is convenient for better readability.

## Technical Validation

### Glucose readings

We assessed the agreement of measurements made by bedside fingerstick tests versus those made in the laboratory. Pairing the first readings of both methods was not always possible (*n* = 216) because during that ICU stay only one reading is recorded or only one method was used to assess glycemia. In the 11,904 paired readings, the predominant gender for this analysis is male (*n* = 6,922 pairs) and females represent nearly 42% (*n* = 4,982 pairs). Further, in 10,488 ICU stays the patient survived and the remaining 1,416 died in the ICU. The lengths of ICU stay are between 129.8 minutes and 104.2 days with a mean of 296.3 ± 316.3 hours. SOFA scores are between 0 and 21 units with a mean of 4.7 ± 3.3. Also, age is still within the Q_1_ and Q_3_ of the entire dataset^3^.

The graphs in Fig. 2 is shown the Bland-Altman plots in four different time-gaps. Values of Δ Glucose <−400 mg/dL were not included to facilitate the comparison of all plots. They show that the bias for tests temporally close together is relatively small. For instance, bias is about 2 points on average when the tests are conducted within 30 minutes. As seen in Fig. 2, when the tests were measured in close temporal proximity, particularly when the average glucose is <200 mg/dL, the average difference between fingerstick and laboratory-derived measurements is quite small.

Wilcoxon rank test in each time-gap for ∆ glucose demonstrated that there is a difference between both methods at 5% of significance. The results for each time-gap (time ranges expressed in minutes) are **[0, 30)** median ∆ glucose = 2.0 mg/dL, Q_1_ – Q_3_ [−17.0, 21.0], P = 0.024; **[30, 60)** median ∆ glucose = 4.0 mg/dL, Q_1_ – Q_3_ [−4.0, 26.0], P < 0.001; **[60, 120)** median ∆ glucose = 0.0 mg/dL, Q_1_ – Q_3_ [−2.0, 18.0], P < 0.001; **[120, 26,748]** median ∆ glucose = 15.0 mg/dL, Q_1_ – Q_3_ [−13.0, 44.5], P < 0.001. Although statistically significant, the median difference between tests, particularly over the shortest time periods are unlikely to be clinically significant for the vast majority of patients.

In Table 6, we report the factors associated with having more fingerstick tests than laboratory results over an ICU-stay. In a multivariable logistic regression analysis, we found that older age, male gender, and being diabetic were independently associated with a statistically significant higher frequency of fingerstick tests. Furthermore, emergency admissions and in-hospital mortality were associated with more laboratory tests, also statistically significant. No statistically significant associations between ethnicity or severity of illness (SOFA) were found at the 0.05 level.

**Table 6:** Logistic regression results and factors associated with having more fingerstick glucose readings than lab analyzed tests. (*n* = 11,904 paired readings).

Variable	Adjusted Odds Ratio (AOR)	95% Confidence Interval for AOR	*p*-values
Age (per year increase)	1.00	1.00–1.01	0.004
Admission type (Emergency)	0.56	0.50–0.63	<0.001
Ethnicity (White)	0.98	0.90–1.08	0.738
Gender (Male)	1.15	1.06–1.24	0.001
Mortality in-hospital	0.53	0.47–0.60	<0.001
SOFA (per point increase)	1.01	0.99–1.02	0.368
Diabetes	1.82	1.67–1.98	<0.001

### Pairing of glucose readings and insulin inputs

The frequency of each rule during the process of alignment (how many times these rules were applied) was determined and presented in the Methods section. The rates of pairing are presented in Table 3. The highest rate of pairing was seen with boluses of short-acting insulin (80.5%). The highest rate of misalignment was noted with injections of intermediate-acting insulin (75.7%), followed by long-acting insulin (66.0%). The rules likely failed to capture an associated glucose value because medium and long-acting insulin administrations may be guided by estimates of basal glucose control such as morning pre-breakfast glucose or patient weight, rather than dictated by a glucose reading at the time of insulin administration^16^.

Table 7 further describes the results of the pairing when stratifying by type of insulin. Boluses of short-acting were associated with the highest mean (187.7 mg/dL) and median (173.0 mg/dL) glucose values compared to infusions of short-acting insulin. The latter were associated with the lowest mean (166.9 mg/dL) and median (145.0 mg/dL) glucose values. Long-acting insulin had the largest range of insulin values (180 Units) as referred to in Table 7.

**Table 7:** Overview of insulin inputs that were gathered while in the ICU and aligned with a glucose reading after applying the proposed rules.

	Mean ± SD	Min	Max	Q_1_	Median	Q_3_
**Infusions of short-acting insulin**						
Glucose readings (mg/dL)	166.9 ± 74.1	90.0	847.0	122.0	145.0	184.0
Insulin (U/hour)	4.9 ± 4.1	3 × 10^−3^	29.8	2.0	4.0	6.0
**Boluses of short-acting insulin**						
Glucose readings (mg/dL)	187.7 ± 58.2	90.0	901.0	150.0	173.0	211.0
Insulin (U)	4.5 ± 3.0	0.1	17.0	2.0	4.0	6.0
**Boluses of intermediate-acting insulin**						
Glucose readings (mg/dL)	172.6 ± 66.5	90.0	481.0	126.5	151.0	202.0
Insulin (U)	18.6 ± 15.3	1.0	180.0	8.0	15.0	25.0
**Boluses of long-acting insulin**						
Glucose readings (mg/dL)	153.6 ± 65.0	90.0	543.0	111.0	130.0	175.0
Insulin (U)	23.4 ± 16.1	1.0	180.0	10.0	20.0	30.0

**Fig. 3:**
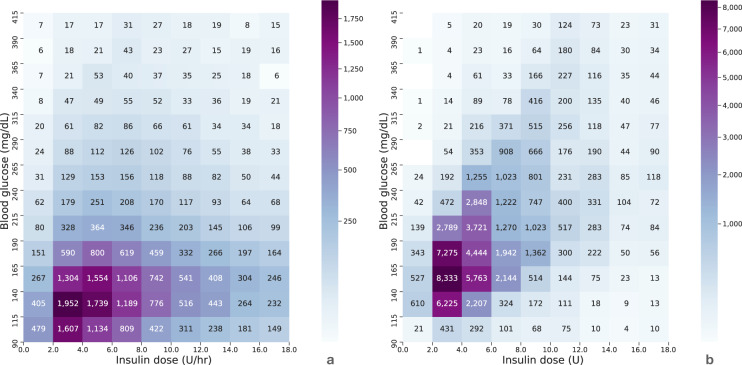
Preceding glucose readings paired with an insulin input. (**a**) Previous glucose readings of patients admitted to the ICU when an infusion of short-acting insulin administered (IV) starts, or changes infusion rate, or stops (*n* = 28,891). (**b**) Previous glucose readings when short-acting insulin boluses were administered (*n = *70,951). Glucose readings ≥415 mg/dL were not included in both plots to compare. Each cell represents the number of insulin - glucose pairs that fall within the specified range indicated along each axis.

The insulin-glucose alignments were displayed graphically to clinically validate the results. Figure 3a plots the density of each insulin infusion event aligned with a preceding glucose reading. As serum glucose values rise, there is a trend toward increasing doses for infusions of insulin. The most frequent dose rate was 2.0–4.0 Units per hour to treat glucose values within a range of 115.0–140.0 mg/dL (1,952 events).

Figure 3b plots the density of each glucose-insulin alignment for short-acting boluses. This density plot also follows the general practice of treating higher glucose values with higher insulin doses. The notable highlight of this figure is that the most frequently administered dose of short-acting insulin was 2–4 Units to treat glucose values in the range of 140–165 mg/dL (8,333 occurrences). Comparing plots in Fig. 3, there is noticed that a higher density of doses for infusions were located in lower glucose readings compared to when boluses of short-acting insulin were administered.

To complement this analysis, and to show the difference between implementing and not implementing the proposed rules, two additional cohorts were created to compare with the pairing criteria proposed above. The results of these comparisons are described in Table 8. Table 8 shows the glucose values paired to a bolus of insulin for a more liberal alignment window of 90 minutes as proposed previously (scenario **A**), a more conservative time window of 60 minutes (scenario **B**, additional cohort 1), and no time window adjustment (scenario **C**, additional cohort 2). With fewer rules or constraints, a greater amount of data was gathered, in terms of the absolute number of entries. It is expected that an insulin event would pair with a glucose reading more frequently in the non-adjusted subset because it was subject to fewer constraints than the two adjusted subsets. In other words, the non-adjusted subset (**C**) applies more simple criteria for pairing insulin events and glucose readings.

**Table 8:** Results after pairing boluses of short-acting insulin with a preceding glucose reading.

Scenario	Pairing rules	Paired glucose readings and boluses of short-acting insulin								
		Patients	Boluses Paired entries (%)		Mean ± SD	Min	Q_1_	Median	Q_3_	Max
A	Applied (90 min)	9,370	88,460	mg/dL	187.7 ± 58.2	90.0	150.0	173.0	211.0	901.0
			71,455 (80.8)	U	4.5 ± 3.0	0.1	2.0	4.0	6.0	17.0
B	Applied (60 min)	9,370	88,460	mg/dL	187.7 ± 58.1	90.0	150.0	173.0	211.0	901.0
			64,925 (73,4)	U	4.5 ± 3.0	0.1	2.0	4.0	6.0	17.0
C	Not applied	9,370	88,460	mg/dL	183.0 ± 58.3	2.0	145.0	169.0	207.0	901.0
			78,881 (89.2)	U	4.5 ± 3.0	0.1	2.0	4.0	6.0	17.0

When no rules are considered, 89% alignment was achieved. Alignment percentage decreases as the maximum allowed time gap between an insulin event and glycemic check decreases. The most notable difference among these subsets was in the range of glucose readings paired with an insulin administration. Specifically, glucose values below 90 mg/dL (as low as 2 mg/dL) were paired with a bolus of regular insulin in the non-adjusted subset. This does not reflect what happens in the ICU. This pairing error can lead to misleading conclusions if used to generate further insights.

In Table 9, the statistical analysis and comparison of scenarios **A** vs **C** and **B** vs **C** are presented. For both comparisons in all the periods, the null hypothesis was rejected. This demonstrated that the samples paired using the proposed rules were different than those paired when the rules were not applied. Moreover, the median difference between scenarios and percentiles (Q_1_ and Q_3_) might be clinically relevant for the vast majority of patients, especially over the largest time-gaps.

**Table 9:** Hypothesis testing to compare pairing scenarios.

Scenarios to compare	Time-gap (Δ*θ*) between insulin event and glycemic check					
	Minutes elapsed	[0, 30)	[30, 60)	[60, 90)	[90, 120)	[120, 21,176)
B vs C	Median Δ glucose	0	0	157	171.5	94
	Q_1_–Q_3_	0–0	0–0	0–196	137–210	31–169
	*p*-value	<0.001	<0.001	<0.001	<0.001	<0.001
	Mean ± SD	1.6 ± 13.1	2.3 ± 12.8	132.1 ± 107.0	172.7 ± 86.1	106.4 ± 84.3
	Δ glucose = 0 (instances)	4,304	2,369	383	0	13
	Total instances	4,454	2,547	1,312	502	1,792
A vs C	Median Δ glucose	0	0	0	157.5	86
	Q_1_–Q_3_	0–0	0–0	0–0	44–195	29–166
	*p*-value	<0.001	<0.001	<0.001	<0.001	<0.001
	Mean ± SD	2.2 ± 16.8	2.4 ± 13.5	4.5 ± 16.6	168.2 ± 86.0	103.4 ± 83.7
	Δ glucose = 0 (instances)	4,304	2,369	1,143	48	13
	Total instances	4,468	2,548	1,313	502	1,792

In the last time-gap, there were 13 instances in our analysis where the recorded value of the first glucose reading was the same in all scenarios. However, the recording time was completely different compared to scenario C. This demonstrates that the curation process should be carried out with well define curation rules to gather accurate information for our analysis. Without careful and thoughtful curation of a dataset, subsequent analysis will inevitably provide misleading or flawed models.

The main difference between scenario B and C was in the amount of information that was gathered to use for future analysis. Analysts should evaluate what is the best time-gap for the study they want to perform and how much information they want to keep. The more information available, the more knowledge is available to feed into a model or statistical analysis. Thus, researchers should decide which timeframes adapt the best to the aim of their studies.

This manual team-based approach provides distinct advantages to automated methods commonly used in data science to detect outliers and erroneous values (e.g.: statistical approaches based on parametric and non-parametric methods). The main advantage of automated methods is that they are less time consuming than the one proposed in this work. However, they may lead to inaccurate, or worse, biased models if flawed assumptions were encrypted during the data preparation. The team of data scientists and domain experts can iteratively interrogate the data and create rules to maximize fidelity to the clinical context. In addition, careful curation of the data must take into consideration various types of errors that are associated with data entry by clinical staff, which requires familiarity with the workflow. For instance, when a drug is administered, the data entry timestamp (store time) might be preceding the administration timestamp (chart time); or manual entry of different labels of the same drug that creates several items ids associated with the drug. Thus, this process requires the careful creation of rules for processing data that balances the loss of the least amount of information with the removal of inaccurate data.

## Usage Notes

The data that support the findings of this paper employs the MIMIC-III data, a publicly available dataset^3^ that has been widely used in the analysis of real-world health data^17^. The access to these services is managed directly by PhysioNet^14^ and researchers should request access by themselves. They must sign a data use agreement, which outlines the data usage and security standards and prohibits any effort to identify the patients in MIMIC. This database is available as a collection of *comma separated value* (CSV) files. Further instructions to import them into different database systems are available on the MIMIC website (https://mimic.physionet.org/gettingstarted/dbsetup/).

The data subset copies are available in PhysioNet^14^ under the project name “*Curated data for describing the blood glucose management in the intensive care unit*”^15^. As an alternative, the 1_0_ara_curation_I.ipynb notebook details how to build the data subsets from the MIMIC-III tables. The 2_0_ara_pairing_II.ipynb notebook contains a hand-on example that shows how both glucose readings and insulin boluses can be paired with the rules proposed in this work.

The queries within the notebooks are optimized to be used on Google’s BigQuery (SQL standard). The two JUPYTER notebooks can be run on Google’s Colaboratory. In case the user runs these notebooks locally, the user should have installed JUPYTER notebooks and required dependencies and modules. The Live Script requires at least MATLAB version R2019b. Alternatively, HTML copies of these Live Scripts are shared in the folder Notebooks (see Code availability).

The assumptions and rules, and their implementation, are provided in detail to serve as a starting point for further refinement of the queries and subsequent analysis. While this study focused primarily on the management of hyperglycemia, the analysis of circumstances surrounding hypoglycemia is limited. Low glucose values were accounted for in the process of insulin pairing and tracking insulin infusion discontinuation. However, there remains an opportunity to analyze in more detail the management of hypoglycemia in the ICU. For example, tracking the glucose values that led to the decision to stop an insulin infusion would provide useful information to describe real-world practice. The authors hope that this curated dataset will be used to further investigate trends in ICU glucose control and how relate to patient outcomes.

## Data Availability

All code used for data extraction, processing, and visualization is available online (two JUPYTER notebooks in Python 3.7) and two MATLAB’s Live Script files) in the PhysionNet project associated with the present paper^15^. These scripts are publicly available to allow for reproducibility and code reuse. The associated PhysioNet project contains 3 folders. The Data folder contains the data subsets described in this paper. The content of the other folders is explained below. ***Queries folder*** Contains the queries to obtain the raw insulin entries and blood glucose readings. The queries can be run on Google’s BigQuery. The file glucose_readings.sql contains the code to extract glucose readings, and the file insulin.sql the codes for insulin entries. ***Notebooks*** Contains the following files: JUPYTER notebooks 1.0-ara-data-curation-I.ipynb: This notebook contains the processing stages to obtain the curated entries of glucose readings and insulin inputs. 2.0-ara-pairing-II.ipynb: This notebook contains the pairing rules to link a preceding glucose reading with a regular insulin input. MATLAB Live Scripts Glucose_Analysis.mlx: This contains a deeper statistics analysis on the glucose readings. It is a complementary analysis for 1.0-ara-data-curation-I.ipynb notebook. Pairing.mlx: Contains the results related to the pairing of a preceding glucose reading and an insulin event. It is a complementary analysis for the 2.0-ara-pairing-II.ipynb notebook. Glucose_Analysis.html & Pairing.html: Contain the same information as the scripts mentioned above, but readable in a web browser. Functions subfolder: Contains MATLAB functions that are called in the Live Scripts described above.
